# Clinical monitoring: infliximab biosimilar CT-P13 in the treatment of Crohn’s disease and ulcerative colitis

**DOI:** 10.3109/00365521.2016.1149883

**Published:** 2016-03-22

**Authors:** Radan Keil, Martin Wasserbauer, Zdena Zádorová, Jan Hajer, Pavel Drastich, Pavel Wohl, Marek Beneš, Martina Bojková, Pavel Svoboda, Michal Konečný, Přemysl Falt, Tomáš Vaňásek, Martin Pešta, František Pešek, Luděk Bouchner, Jana Koželuhová, Aleš Novotný, Lucie Bartůsková, Julius Špičák

**Affiliations:** ^a^ Department of Internal Medicine, 2nd Faculty of Medicine, Charles University in Prague and Motol University Hospital, Prague, Czech Republic; ^b^ 2nd Department of Internal Medicine, 3rd Faculty of Medicine, Charles University in Prague, FNKV, Prague, Czech Republic; ^c^ Department of Hepatogastroenterology, Institute for Clinical and Experimental Medicine, Prague, Czech Republic; ^d^ Clinic of Internal Medicine, University Hospital Ostrava, Ostrava, Czech Republic; ^e^ 2nd Department of Internal Medicine, University Hospital Olomouc, Olomouc, Czech Republic; ^f^ Digestive Diseases Center, Vítkovice Hospital, Ostrava, Czech Republic; ^g^ 2nd Department of Internal Medicine-Gastroenterology, University Hospital Hradec Králové, Hradec Králové, Czech Republic; ^h^ Department of Internal Medicine, Hospital Na Bulovce, Prague, Czech Republic; ^i^ 2nd Department of Internal Medicine, University Hospital Plzeň-Bory, Plzeň, Czech Republic; ^j^ 4th Department of Internal Medicine – Clinic of Gastroenterology and Hepatology, The General University Hospital in Prague, 1st Medical Faculty, Charles University in Prague, Prague, Czech Republic; ^k^ Department of Economic and Social Policy, University of Economics, Prague, Czech Republic

**Keywords:** Biosimilar, CT-P13, inflammatory bowel disease, infliximab

## Abstract

**Objective:** The infliximab biosimilar CT-P13 (Remsima^®^, Inflectra^®^) was approved in Europe for the treatment of inflammatory bowel disease (IBD) based on extrapolation of data from patients with rheumatic disease. Because there are limited published reports on clinical outcomes for IBD patients treated with CT-P13, we monitored responses to induction treatment with this biosimilar in patients with Crohn’s disease (CD) or ulcerative colitis (UC) in centres across the Czech Republic.

**Material and methods:** Fifty-two patients with CD (*n* = 30) or UC (*n* = 22) were treated with 5 mg/kg CT-P13 for up to 14 weeks. Effectiveness of therapy was evaluated with the Crohn’s Disease Activity Index (CDAI) or the Mayo Scoring System (MSS) in patients with CD or UC, respectively, before and after 14 weeks. Additional goals were to evaluate weight changes, serum C-reactive protein (CRP) levels, and complications/adverse events.

**Results:** In patients with CD, remission (CDAI <150) was achieved in 50.0% of cases, and partial response (≥70-point decrease in CDAI score from baseline) in the remaining 50.0%. In patients with UC, remission (total score on partial Mayo index ≤2 points) was achieved in 40.9% of cases, partial response (≥2-point decrease in partial Mayo score from baseline) in 54.5%, and no response in 4.5%. There were statistically significant improvements in CDAI, MSS and CRP serum levels after 14 weeks of therapy, and body weight increased. Four adverse events were identified (*n* = 1 each): lower-extremity phlebothrombosis, herpes labialis, pneumonia and allergic reaction.

**Conclusions:** This prospective observational study provides evidence of the effectiveness of CT-P13 in IBD.

## Introduction

Inflammatory bowel disease (IBD), including ulcerative colitis (UC) and Crohn’s disease (CD), is a chronic inflammatory disorder of the gastrointestinal tract.[1,2] Activation of T cells and a broad spectrum of inflammatory mediators, especially tumour necrosis factor (TNF), results in pathological inflammation of the intestinal mucosa and plays an essential role in the pathogenesis of IBD.[1–3] The interference of this inflammatory pathway with biological therapies has revolutionised the treatment of IBD. Infliximab, the first anti-TNF agent, is a chimeric monoclonal antibody (comprising 75% human and 25% murine sequences), which has a high specificity and affinity to TNF, thereby neutralising its activity. However, while anti-TNF therapy provides undeniable benefits to patients’ health,[4] it also significantly increases the cost of treatment.

Biosimilars are products that are highly similar to their originator biological drug, or ‘reference medicinal product (RMP)’. However, biosimilars are not the same as generic versions of small-molecule drugs. Generics have relatively simple chemical structures and can thus be manufactured to be identical to their originator drug. In contrast, biological drugs are large, structurally complex proteins produced in living systems. Therefore, it is not possible for biosimilars to be completely identical to their RMP. Comprehensive and extensive comparability programmes are required by regulatory authorities for biosimilar approval.[5] This comparability assessment includes information on product quality, as well as non-clinical and clinical data. Biosimilars represent an opportunity to reduce health care costs, while offering a similar level of efficacy and safety to that of their RMPs.[5] Currently, around 20 biosimilars are approved by the European Medicines Agency (EMA).[6]

The infliximab biosimilar CT-P13 (Remsima^®^, Inflectra^®^) is now available for clinical use in CD and UC in many countries, including those of the European Union. CT-P13 was approved in these countries in all indications held by the RMP, based on a comprehensive non-clinical comparability exercise and on the extrapolation of clinical data from two rheumatology studies called PLANETAS and PLANETRA.[7,8] Some concerns regarding the use of CT-P13 in gastroenterological indications have been raised, especially because of differences in dosing and concomitant immunosuppressive therapy between these indications and rheumatological diseases. To date, there are relatively few published reports regarding clinical outcomes achieved with CT-P13 in patients with IBD.

The main goal of this observational prospective study was to evaluate the effectiveness of CT-P13 in terms of response to induction treatment (remission, partial response or no response) in patients with CD or UC after 14 weeks of therapy (measured after the final induction dose of CT-P13 at Week 14). The effectiveness of therapy was evaluated individually using the Crohn’s Disease Activity Index (CDAI) in patients with CD or the Mayo Scoring System (MSS) in patients with UC, and also by endoscopy and C-reactive protein (CRP) values, after 14 weeks of therapy. Additional goals of the study were to evaluate the weight profile of patients during therapy, as well as any complications or adverse effects of treatment.

## Materials and methods

### Patients

Clinical monitoring was performed at the Gastroenterology and Hepatology Departments of 10 medical centres across the Czech Republic. Patients with CD or UC were eligible to participate in the study if they met the indication criteria for CT-P13 as per the Summary of Product Characteristics for this biological agent in the Czech Republic. At the time of study initiation, CT-P13 was indicated for treatment of moderate to severe active CD or fistulising active CD in adult patients who had not responded despite a full and adequate course of therapy (corticosteroid, antibiotic and immunosuppressive therapy), who were intolerant to these types of therapies, or who had medical contraindications for these therapies. CT-P13 was also indicated for treatment of moderate to severe active UC in adult patients who had an inadequate response to conventional therapy (corticosteroids, 6-mercaptopurine and azathioprine), were intolerant to these types of therapies, or who had medical contraindications for these therapies.

As per the indications for CT-P13, patients were allowed to use other treatments for IBD before biological therapy. Patients could also receive concomitant therapy during CT-P13 treatment. In order to ensure that results were not affected by potentially confounding factors, exclusion criteria were as follows: previous anti-TNF treatment, complications of IBD leading to surgery (strictures, abscess or perforations), and contraindication(s) for anti-TNF treatment. All patients were examined at a Pneumology Department before initiation of the therapy. The first patient was enrolled in January 2014.

### Ethical statement

Each patient provided signed consent to therapy with CT-P13 (a registered medicine in the Czech Republic) prior to being accepted for this study.

### Treatment and study parameters

Eligible patients (men and women) with CD or UC were assigned to biological treatment with CT-P13 (5 mg/kg intravenous infusions at Week 0, 2, 6 and 14), according to the indication criteria listed in the Summary of Product Characteristics in the Czech Republic. Patients could continue on therapy after Week 14 but data on treatment beyond Week 14 are not presented here.

Patients were asked to answer some basic questions at the beginning of the monitoring in order to collect information on baseline demographics and characteristics. The observed parameters (CDAI in patients with CD, MSS in patients with UC, CRP, weight and endoscopic findings) were assessed before CT-P13 therapy began and after 14 weeks of therapy. Every patient also underwent endoscopy before study enrolment.

In patients with CD, a partial response was defined as a ≥70-point decrease from baseline in CDAI score and remission was defined as a CDAI score of <150. In patients with UC, a partial response was defined as a ≥2-point decrease from baseline in partial Mayo score and remission was defined as a total score on the partial Mayo index of ≤2. Patients who did not show a partial response or remission were considered non-responders.

### Statistical analyses

Standard summary statistics were used to describe primary data; median supplemented by minimum and maximum was used for cardinal data, absolute and relative frequencies for nominal variables. The statistical significance of differences between men and women was analysed using a Mann–Whitney *U* test (cardinal data) and Fisher’s exact test (nominal variables). Statistical significance of time-related changes in pair-wise comparisons was analysed using the Wilcoxon paired test for detailed comparisons of two time points. The value α = 0.05 was adopted as a level of statistical significance in all analyses. Analyses were performed using SPSS 22 (IBM Corporation, 2013).

## Results

### Patient demographics and disposition

Fifty-two eligible patients (29 men and 23 women) who had been diagnosed with IBD (30 with CD, 22 with UC) were enrolled (Table 1). Patients were between 18 and 71 years of age, and the median age before biological treatment of IBD was 37 years for men and 39 years for women. The median age of patients at first diagnosis of IBD was 30.0 years for men and 28.5 years for women (Table 1). Affected areas in patients with CD were small intestine (23 patients), colon (22 patients), perianal fistula (9 patients) and other types of fistula (2 patients) (Table 1). Only the colon was affected in patients with UC. In the UC group before enrolment, pancolitis was present in 12 patients, left-sided colitis in 9 patients, and proctitis in 1 patient. Montreal classification status was noted in all patients (CD and UC groups) prior to enrolment (Tables 2 and 3).

**Table 1.** Baseline patient demographics and clinical characteristics.

Number of patients (men/women)		
CD	30 (14/16)	
UC	22 (15/7)	
	Men	Women
Age before therapy, median (range)	37 (19.0–71.0)	39 (18.0–61.0)
Age at diagnosis, median (range)	30 (14.0–58.0)	28.5 (13.0–55.0)
Disease activity, median (range)		
Mayo score	4 (0.0–10.0)	8 (0.0–9.0)
CDAI	186 (0.0–345.0)	283 (0.0–400.0)
CRP, median (range)	28 (0.0–85.0)	11 (0.0–57.0)
Weight, median (range)	80 (55.0–168.0)	63 (45.0–97.0)
Site of disease in CD, *n* (%)	All patients	
Small intestine	23 (76.7)	
Colon	22 (73.3)	
Perianal fistula	9 (30.0)	
Other types of fistula	2 (6.7)	
Previous treatment, *n* (%)		
5-aminosalicylates	47 (90.4)	
Oral corticosteroids	46 (88.5)	
Azathioprine	39 (75.0)	
Others	10 (19.2)	
Concomitant treatment, *n* (%)		
5-aminosalicylates	40 (76.9)	
Oral corticosteroids (low dose)	14 (26.9)	
Azathioprine	29 (55.8)	
Others	4 (7.7)	

CD: Crohn’s disease; CDAI: Crohn’s Disease Activity Index; CRP: C-reactive protein; UC: ulcerative colitis.

**Table 2.** Montreal classification in CD patients at enrolment (total number of patients = 30).

	*n*
A – age at diagnosis, years	
A1 (<16)	2
A2 (17–40)	20
A3 (>40)	8
L – localisation at diagnosis	
L1 (ileal)	3
L2 (colonic)	5
L3 (ileocolonic)	22
L4 indicator (upper gastrointestinal tract)	1
B – behaviour	
B1 (nonstricturing, nonpenetrating)	22
B2 (stricturing)	6
B3 (penetrating)	2
p indicator (perianal disease)	9

CD: Crohn’s disease.

**Table 3.** Montreal classification in UC patients at enrolment (total number of patients = 22).

	*n*
E – extent	
E1 (proctitis)	1
E2 (left-sided colitis)	9
E3 (pancolitis)	12
S – severity	
S0 (clinical remission)	0
S1 (mild)	3
S2 (moderate)	17
S3 (severe)	2

UC: ulcerative colitis.

Initial symptoms of disease were diarrhoea (reported by 50 patients), difficult defaecation (8 patients) and other symptoms (14 patients). Finally, extraintestinal manifestations of IBD were reported by 16 patients (skin was affected in 2 patients, joints in 13 patients and 1 patient reported other extraintestinal manifestations).

Prior to biological therapy with CT-P13, 47 patients had received 5-aminosalicylates, 46 oral corticosteroids, and 39 azathioprine; other therapy was used in 10 patients. In terms of concomitant therapy during CT-P13 treatment, 40 patients were treated with 5-aminosalicylates, 14 with low-dose systemic corticosteroids, 29 with azathioprine, and 4 with other therapy (Table 1).

The majority of patients received 5 mg/kg intravenous infusions of CT-P13 at Week 0, 2, 6 and 14 (eight patients received only three doses of the therapy). Two of the 52 enrolled patients (both in the UC group) discontinued therapy prior to Week 14; one because of allergic reaction and one because of inefficiency of the therapy after the third dose (this patient also suffered with pneumonia).

### Effectiveness in patients with CD

In the CD group (*n* = 30), all patients achieved either remission (*n* = 15) or partial response (*n* = 15) after 14 weeks of therapy. In patients who achieved remission, the most noticeable effect was observed after the second dose of therapy. In patients who showed partial response, the most noticeable effect was observed after the third dose of therapy.

**Figure 1.**
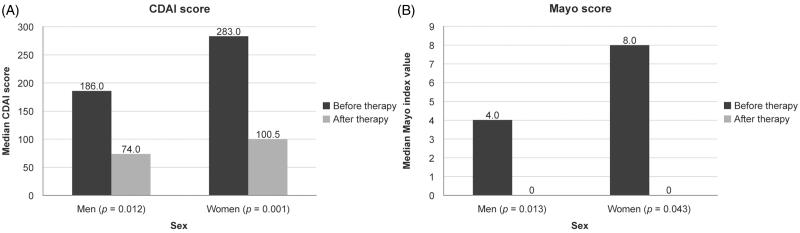
Median disease activity scores at baseline and after 14 weeks of treatment with CT-P13. (A) Crohn’s Disease Activity Index (CDAI) score in patients with Crohn’s disease. (B) Mayo score in patients with ulcerative colitis.

The median CDAI value in the CD group before therapy was 186.0 for men and 283.0 for women and this decreased to 74.0 (*p* = 0.012) and 100.5 (*p* = 0.001), respectively, after 14 weeks of therapy (Figure 1A). CT-P13 treatment in patients with fistulas resulted in both clinical and laboratory improvements, demonstrated by a reduction in fistula activity and a decrease in CRP levels, respectively.

### Effectiveness in patients with UC

In the UC group (*n* = 22), remission was achieved in nine patients, and partial response was observed in 12 patients after 14 weeks of treatment. One patient showed no response to therapy. In patients who achieved remission, the most noticeable effect was observed after the second dose of therapy. In patients with partial responses, the most noticeable effect was observed after the third dose of therapy.

The median MSS value in the UC group before therapy was 4.0 for men and 8.0 for women, and this decreased to 0.0 in both men and women (*p* = 0.013 and *p* = 0.043, respectively) after 14 weeks of therapy (Figure 1B).

### Effectiveness in all patients as measured by CRP levels (CD and UC groups combined)

**Figure 2.**
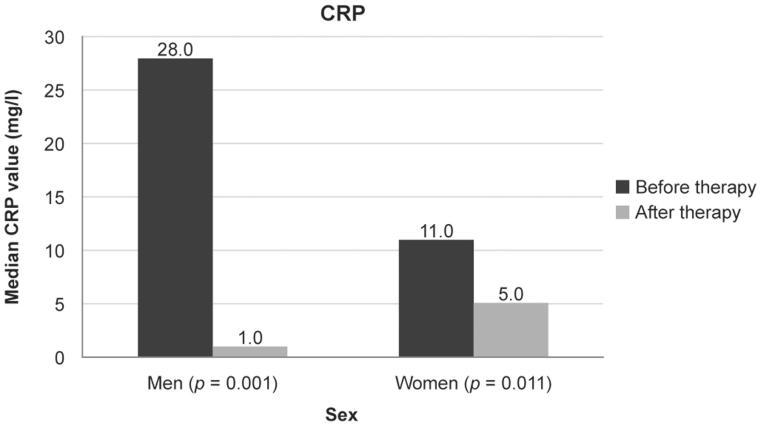
Median levels of C-reactive protein (CRP) at baseline and after 14 weeks of treatment with CT-P13 in patients with inflammatory bowel disease.

The median CRP level was 28.0 mg/L for men and 11.0 mg/L for women before therapy and this decreased to 1.0 mg/L for men (*p* = 0.001) and 5.0 mg/L for women (*p* = 0.011) after 14 weeks of therapy (Figure 2).

### Weight changes

**Figure 3.**
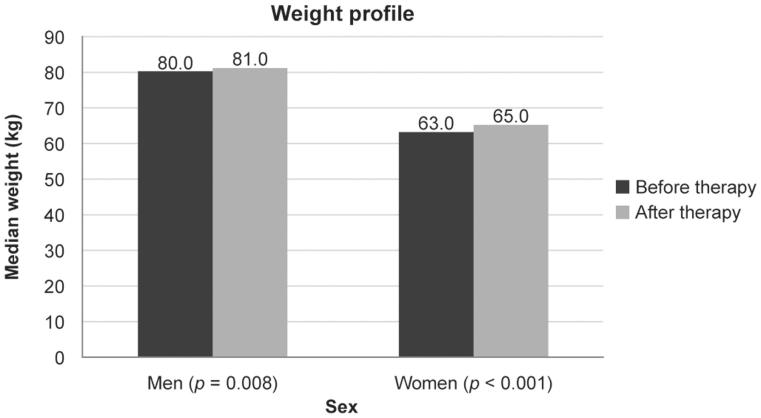
Median weight at baseline and after 14 weeks of treatment with CT-P13 in patients with inflammatory bowel disease.

Median weight before the therapy was 80.0 kg in men and 63.0 kg in women. This increased to 81.0 kg (*p* = 0.008) and 65.0 kg (*p* < 0.001), respectively, after 14 weeks of therapy (Figure 3).

### Safety and tolerability

Four complications were identified during the therapy: phlebothrombosis of the lower extremity (one patient), herpes labialis (one patient), pneumonia (one patient) and an allergic reaction (one patient).

## Discussion

Infliximab is well established for the treatment of moderate to severe active CD that is refractory to conventional treatment,[9,10] and for the treatment of fistulising disease.[11] Infliximab is also used for patients with moderate to severe active UC who have an inadequate response to conventional therapy.[12–14]

In our group of patients with CD treated with the infliximab biosimilar CT-P13, remission was achieved in 50% of cases and partial response in the other 50%. In the group of patients with UC treated with CT-P13, remission was achieved in 40.9% of cases, partial response in 54.5% and there was no response in 4.5%. In line with these findings, a Korean study of 110 IBD patients treated with CT-P13 (CD, *n* = 59 and UC, *n* = 51) reported that clinical remission rates of 77.3% and 47.8% were achieved in TNF-antagonist-naive CD and UC patients, respectively, after 30 weeks of treatment.[15] Several reports from other observational studies in countries including South Korea, Hungary, Poland and Norway now also support the use of CT-P13 in IBD patients.[16–22]

The effectiveness of infliximab RMP in patients with IBD has been demonstrated in many studies. A response rate at Week 4 of 81% was seen in patients with CD after treatment with 5 mg/kg infliximab RMP, compared with 17% in the placebo group; 48% of patients treated with infliximab RMP and 12% of patients in the placebo group showed a response at Week 12.[9] In another study involving patients with CD, 89% of patients showed a response after induction therapy with infliximab RMP.[23] There are also published studies evaluating the effectiveness of infliximab RMP in UC, including ACT 2, which has reported Week 8 response (clinical remission) rates of 29.3% for patients in the placebo group and 64.5% for patients treated with 5 mg/kg infliximab RMP.[13] A reduced need for colectomy in patients with UC who were treated with infliximab RMP has also been reported.[12]

In addition to the response data collected in the current study, our patients also showed a significant decrease in CRP values during CT-P13 treatment. This measure closely correlates with CD and UC disease activity.[24,25] Furthermore, in our patients, statistically significant decreases in CDAI and MSS were seen, compared with baseline. CDAI in patients with CD and MSS in patients with UC are classification systems of IBD activity and severity, which are used worldwide and which also influence the management of patients.[26,27]

An additional goal of this study was to monitor the weight profile of patients during therapy with CT-P13. Both sexes showed a significant weight gain after Week 14. IBD affects nutritional status in patients and weight loss is a fundamental symptom of the disease.[28,29] Indeed, weight loss is seen in about 60% of patients with CD before diagnosis [27] and is also a marker of disease activity used in calculating the CDAI. Again, our findings with respect to weight are aligned with historical results with infliximab RMP. Treatment of CD with infliximab RMP has been reported to result in weight gain [30,31] and also to improve growth and bone health in paediatric patients with IBD.[32]

Treatment with an anti-TNF agent is relatively safe if used for appropriate indications [27] but can be associated with potentially serious adverse effects. Four complications occurred in our group of patients during treatment; however, it cannot be confirmed whether these were treatment-related. Two of these complications were classified as infectious events: pneumonia and herpes simplex. The development of pneumonia during treatment with infliximab RMP has been described in the literature.[33] In a recent CT-P13 study, serious infectious adverse events occurred in 5.7% of all patients.[22] In the current study, pneumonia together with an inadequate effect of the biosimilar after the third dose led to termination of therapy by one patient. Another patient was affected by deep vein thrombosis and one experienced an allergic reaction after infusion of CT-P13, which led to the termination of use by this patient. Acute infusion reactions are also associated with the use of infliximab RMP.[26,27,33] These reactions can have different frequencies and severities. One infusion centre reported that infusions of infliximab RMP caused at least one infusion reaction in 9.7% of patients and that 6.1% of infusions were complicated by infusion reactions.[34] Infusion-related reactions were present in 6.7% [22] and 5.2% [35] of patients treated with CT-P13 in two recent studies. In our study, this complication presented in 1 of the 52 patients.

Biosimilars are defined by the EMA as biological medicinal products that are similar to authorised biological medicines.[5] CT-P13 is biologically similar to the original infliximab RMP. It is produced in the same type of cell line and has an identical amino acid sequence to infliximab RMP. However, any small difference in the production process (growth conditions, purification process or storage conditions) of a biosimilar may alter the function of this agent.[5,36] Important considerations concern not only the existence of any differences, but also their clinical impact. Both medications must be clinically comparable and interchangeable especially in safety, efficacy and immunogenicity across all indications. Importantly, therefore, randomised controlled clinical studies have been performed to compare the pharmacokinetics (PKs), efficacy and safety of CT-P13 and original infliximab RMP, with these conclusions:

1. The PK profiles of CT-P13 and infliximab RMP were equivalent in patients with active ankylosing spondylitis. CT-P13 was well tolerated by patients, with an efficacy and safety profile comparable to that of infliximab RMP up to Week 30.[7]
2. CT-P13 demonstrated equivalent efficacy to infliximab RMP in patients with rheumatoid arthritis (RA) at Week 30, with a comparable PK and immunogenicity profile. CT-P13 was well tolerated, with a safety profile comparable with that of infliximab RMP.[8]

According to EMA biosimilar guidelines, extrapolation from one indication to another can be considered without the need for additional trials if biosimilarity has been demonstrated by a comprehensive comparability assessment – including efficacy, safety and immunogenicity – in a sensitive indication that is suitable to detect potential clinical differences between the biosimilar and its RMP. This means that a biologically similar drug can be registered for use in all the indications for which it has not been clinically tested, providing it has demonstrated clinical comparability in a key, sensitive indication, and if the original product has been registered for these indications. In this respect, the EMA agreed that RA is the key indication for CT-P13 and approved the extrapolation of CT-P13 across all the same indications as infliximab RMP (including IBD) based on data in this RA patient population. The main benefit of infliximab biosimilars seems to be the reduction of high costs of biological treatment.[5]

Limitations of the current study included a small patient number, and that this was a non-randomised, non-blinded study. However, despite these limitations, our results show positive clinical outcomes following administration of CT-P13 to IBD patients. A multicentre, randomised, double-blind clinical phase 3 study has been initiated to assess safety and non-inferiority of efficacy of CT-P13 and infliximab RMP in adults with CD (ClinicalTrials.gov identifier: NCT02096861). Also, a global registry study for adults or children with active CD and adults with fistulising CD or UC (NCT02326155) has been initiated. The results from these studies are expected to be available in 2016 or 2017.

## Conclusion

In conclusion, with the arrival of biological therapy that selectively blocks the inflammatory cascade, we entered a new era of IBD management. The beginning of the next era in the treatment of CD and UC may be the introduction and use of biosimilars.

Our prospective observational study has provided one of the first sets of evidence of the effectiveness of infliximab biosimilars in the treatment of IBD. However, in the absence of a comparative study, some concerns remain about the equality of CT-P13 and infliximab RMP in the indication of IBD. Therefore, large, double-blind, randomised, prospective studies are needed, and are indeed ongoing, to compare the clinical effectiveness and safety of the biosimilar versus infliximab RMP in this indication.

## Disclosure statement

Dr. Vaňásek reports receipt of personal fees from CELLTRION, Inc. for advisory board participation, outside of the submitted work. All other authors have no financial conflict of interest to declare.

## Funding information

The study was supported by EGIS s.r.o. and Celltrion Healthcare Co., Ltd (only financial support for statistical analysis, language correction and publication charges).
